# Language and Movement Synchronization in Dyadic Psychotherapeutic Interaction – A Qualitative Review and a Proposal for a Classification

**DOI:** 10.3389/fpsyg.2021.696448

**Published:** 2021-10-22

**Authors:** Carl Eduard Scheidt, Stefan Pfänder, Arianna Ballati, Stefan Schmidt, Claas Lahmann

**Affiliations:** ^1^Department of Psychosomatic Medicine and Psychotherapy, University Medical Center, Albert-Ludwigs-University Freiburg, Freiburg, Germany; ^2^Freiburg Institute for Advanced Studies (FRIAS), Albert-Ludwigs-University Freiburg, Freiburg, Germany; ^3^Romanisches Seminar, Albert-Ludwigs-University Freiburg, Freiburg, Germany

**Keywords:** movement synchrony, psychotherapeutic alliance, motion energy analysis, classification of synchrony phenomena, mother-child interaction, coordination acts

## Abstract

In individual psychotherapy verbal communication and movement synchronization are closely interrelated. The microanalysis of timing, rhythm and gestalt of movement has established dynamic movement coordination as a systemic property of the dyadic interaction. Movement synchronization supports and enhances the unfolding of linguistic meaning. In order to substantiate the importance of the concept of synchrony for adult psychotherapy we review evidence from developmental psychology and discuss approaches to measure synchrony with particular reference to the naturalistic setting of dyadic psychotherapy. As the concept of synchrony is still ambiguous, and the respective interactional phenomena are ephemeral and fluid, in the current paper we suggest a set of five criteria for the description of synchronization in general terms and eight additional criteria which specifically enable the description of phenomena of movement synchronization. The five general dimensions are: (1) context, (2) modality, (3) resources, (4) entrainment, and (5) time-lag. The eight categories for the description of movement synchrony are: (1) spatial direction, (2) amplitude, (3) sinuosity, (4) duration, (5) event structure, (6) phase, (7) frequency, and (8) content. To understand the process of participatory sense-making and the emergence of meaning in psychotherapy, synchrony research has to cope with the multimodality of the embodied interaction. This requires an integrated perspective of movement and language. A system for the classification of synchrony phenomena may contribute to the linking of variations and patterns of movement with language and linguistic utterances.

## Introduction: Synchronization at the Intersection of Different Disciplines

The term interactional synchrony refers to the dynamic correspondence of movement and gestalt on various levels of expressive behavior between the participants of an interaction. Originally conceptualized and studied by developmental psychologists, the concept of synchrony has been applied to many different fields of study in the last two decades (Leclere et al., 2014) including interactional linguistics, psychotherapy, cognitive neuroscience and robotics (Delaherche et al., 2012). The growing interest in interactional synchrony has also led to a variety of differing but also partially overlapping concepts and perspectives. In the field of developmental psychology, terms such as imitation and mimicry (Meltzoff and Moore, 1977; Meltzoff and Marshall, 2018) refer to issues of *social learning* (Meltzoff et al., 2009). In a similar vein, the notions of affective resonance (Mühlhoff, 2015), affect attunement (Stern, 1985) and entitativity (Lakens, 2010) point to the *sharing of emotional experience*. In cognitive psychology (Pickering and Garrod, 2004, 2006, 2009) and in cognitive linguistics, (Oben and Brone, 2016) the term alignment refers to synchronization in the use of the same lexical items and equivalent syntactic structures by co-participants in adjacent turns-at-talk. The communication accommodation theory (Giles et al., 2016) accentuates the dynamic process of mutual adaptation in language based on the motivation to decrease social distance.

In psychotherapy research the concept of interactional synchrony has recently received increasing attention. In a review on interpersonal coordination dynamics in psychotherapy Wiltshire et al. (2020) identified four modalities in which interpersonal coordination occurs during psychotherapy: physiology, movement, interpersonal displays, and language/vocalizations. In the reviewed research, movement coordination was most frequently associated with *psychotherapy outcome*, while coordination of the other modalities including language and vocalization were most frequently associated with the therapeutic *alliance* (Wiltshire et al., 2020). Other studies focused on the impact of synchrony on the therapeutic relationship and its potential to deal with ruptures in the therapeutic alliance (Friedman, 2020). The study of synchronization processes at the procedural level of language and non-verbal communication contributes to the understanding of fundamental processes of change in psychotherapeutic interaction.

Here we review current research on synchrony at the interface of movement and language in adult dyadic psychotherapy. Studies on synchronization including movement AND language in psychotherapy are non-extant (Wiltshire et al., 2020). Physiological synchrony, including inter-brain coupling, is a rapidly growing field of research requiring different methodologies (Kleinbub et al., 2020). We focus here on interpersonal synchrony with regards to *movement and language coordination*, in which the process of participatory sense-making (De Jaegher and Di Paolo, 2007) is important. Participatory sense-making refers to the ways in which the intentional activities of the interactants in the psychotherapeutic dialog are coordinated and new meaning is created (Fuchs and De Jaegher, 2009). Movement synchrony is the most intensely studied modality of synchronization in psychotherapy and it is closely related to language in terms of prosody, vocal pitch and linguistic utterances. The methodology, however, for integrating processes of synchronization in these two important modalities is still in its infancy.

As synchrony research in the field of mother-infant observation has had a strong impact on research in adult psychotherapy we start with a brief outline of relevant findings from developmental psychology. We then review measures of synchrony in adult psychotherapy research and finally propose a set of criteria for a classification of synchronization phenomena.

## Definitions of Synchrony

According to Bernieri et al. (1988) the study of synchrony can be divided into three broad perspectives: biological rhythms, simultaneous behavior and perceived synchrony. In the *rhythmical approach* human behavior is understood to occur rhythmically and can be described in terms of cycles, periods, frequencies and amplitudes. From the perspective of *simultaneous behavior* synchrony occurs when one person imitates or mirrors another person’s limb movements or body configuration. *Perceived synchrony* refers to the phenomenological effect of synchrony, that is the perceptual social phenomena (Bernieri et al., 1988). While investigations on biological rhythms and simultaneous behavior are generating objective criteria for identifying coding and quantifying synchronous behavior, research on the phenomenological effects focuses on the subjective and gestalt aspects of synchrony, thereby linking the third- and the first-person perspectives of synchrony research (Bernieri et al., 1988). Notions of affective resonance (Mühlhoff, 2015), affect attunement (Stern, 1985) and entitativity (Lakens, 2010), which point to the *sharing of emotional experience* also refer to the perceptual dimension of the synchrony concept.

The term synchrony is sometimes restricted to forms of movement which are *in-phase*, i.e., the rhythms of the movements between interactants are in identical parts of their cycles and have a phase angle of 0°, whereas the term coordination is used in a superordinate sense for movements which are rhythmically attuned and maintaining *some relative phase with respect to one another* (Cross et al., 2016). The related term of mimicry, according to this definition, refers to the imitation of other’s actions entailing a time-lag (Rennung and Göritz, 2016), whereas interpersonal synchrony refers to movements of two people which overlap in time. In paragraph 5 and 6 we will describe phase and time-lag as important criteria for the description of interpersonal synchrony.

Some authors focus their definition of synchrony on *non-verbal* coordination (Ramseyer, 2010), while others define it on the basis of *linguistic* adjustment processes. Dotter (2014), for example, assumes that coordination is less procedural and more controlled by the matching of complex and differentiated linguistic knowledge systems under the umbrella of linguistic adjustment. Drawing on the research of Schmidt and Herrgen (2011), Dotter (2014) understands synchronization as the adjustment of competence differences in situated linguistic interactions (cf. in a similar vein also Purschke, 2014a, b; Bülow, 2017).

Kim (2015) suggests that there is a common causal mechanism for synchronization. He describes synchrony as a universal phenomenon that has its origins in biopsychological and sociocultural factors that are then differentially shaped according to culture. Kim (2015) applies the concept of synchrony primarily to non-verbal communicative behavior. The synchronization of non-verbal configurations and rhythm reflects the reciprocity of attention, interest and resonance. Indeed, interacting participants might not be consciously aware of what Lakens terms the “entitativity” (Lakens, 2010) produced by synchrony, that is the sense of belonging together in a communicative relationship.

Delaherche et al. (2012) focus on the *temporal dimension* of synchronization: “Synchrony is the dynamic and reciprocal adaptation of the temporal structure of behaviors between interaction partners […] the important element is the timing, rather than the nature of the behaviors” (Delaherche et al., 2012). The authors assume that adjustment processes do not always have to be carried out in exactly the same form, but can take place multimodally using various resources like gaze, gesture and body position. Synchrony does not strictly have to be carried out simultaneously but rather in a certain window of time, which other authors have referred to as the “social present” (Tschacher et al., 2018).

Synchronization is also understood, in accordance with Delaherche et al. (2012), as ephemeral *moments of alertness* and reciprocal concentration (Roth, 2014) in which the participants of the interaction align the temporal structure of (verbal and/or bodily) rhythm and behavior together (Buchholz and Reich, 2014). In moments of synchronization, an experience of mutual understanding as well as a further sense of community and relationship are created (Miles et al., 2009; Wiltermuth and Heath, 2009; Pfänder et al., 2018).

An important distinction, which originated in developmental psychology, concerns the time-scales of the coordination dynamics. Nomikou et al. (2016) suggest to differentiate between two time-scales: one that entails the moment-to-moment adjustments, which are not planned in advance, are constructed online and may be described as a coupling mechanism; and a second time-scale acting on multiple repeated interactions forming the interactants’ expectations about each other’s behavior (Nomikou et al., 2016, 2017). Most research on synchrony in psychotherapy investigates phenomena on the first time-scale.

## Interpersonal Synchrony From the Perspective of Developmental Psychology

The heuristic potential of the concept of synchrony for the analysis of human interaction is largely based on observations of parent-infant interaction. Substantial evidence supports the notion that synchronization is an essential component of the parent-infant relationship from birth onward. During early development, synchrony involves a matching of behavior, emotional states, and biological rhythms between parents and infants (Leclere et al., 2014). Already at three to four months mothers and infants synchronize their behaviors coordinating gaze, facial expressions, orientation patterns and touch (Beebe et al., 2010, 2016). According to Feldman et al. (2011) three main channels of non-verbal synchrony have been identified. *Gaze synchrony* is the matching of social gaze between parent and child, *affect synchrony* refers to the matching of affective expression, which plays an important role in the development of self-regulatory capacities (Cohn and Tronick, 1988) and *vocal synchrony* concerns “proto-conversations,” which serve as building blocks of spoken language and promote attachment security (Jaffe et al., 2001). Each of these forms of synchrony has been shown to predict long-term outcome and is thought to provide essential environmental inputs for physiological and social growth (Feldman et al., 2011).

Common terms referring to synchrony in developmental psychology are mutuality, reciprocity, rhythmicity, harmonious interaction, turn-taking and shared affect. All these terms were originally used to characterize the mother-child dyad (Leclere et al., 2014), but in recent years they have been used to understand processes of interaction and rapport building in one-on-one adult psychotherapy. The key question of “how the largely out-of-awareness process of face-to-face relatedness works, in the implicit/procedural domain of non-verbal behavior” (Beebe and Lachmann, 2020) does not only pertain to mother-infant interactions but also to adult interactions, and specifically to dyadic communication in adult psychotherapy.

Some observations from developmental psychology in this context are of particular importance:

(a) Imitation or mimicry, which are important tools for social learning (Bandura et al., 1963; Meltzoff and Moore, 1983; Meltzoff and Marshall, 2018) and which fulfill a range of adaptive functions (Van Baaren et al., 2003) can be conceived as developmental precursors of synchronization. In the first two years of life, an infant’s imitation becomes increasingly diverse integrating higher-order processes such as goals, intentions, and social context (Arnold and Winkielman, 2020). From a developmental perspective imitation is a core feature of the unfolding capacity for synchronization and the complex integration of transmodal expression.
(b) Interactional synchrony in the mother-infant relationship is associated with a more positive cognitive and psychosocial development. In particular synchrony predicts better adaptation overall [e.g., the capacity for empathy in adolescence (Feldman, 2007)], symbolic play and internal state speech (Feldman and Greenbaum, 1997), the relation between mind-related comments and attachment security (Lundy, 2002, 2003), and mutual initiation and mutual compliance (Rocissano et al., 1987; Lindsey et al., 1997; qtd. in Leclere et al., 2014).
(c) This does not imply, however, that more synchronization is always associated with better outcomes or adaptation. Jaffe et al. (2001) found that mid-range mother-infant and stranger-infant vocal rhythm coordination was optimal for secure attachment at one year of age. High-range synchrony by contrast was associated with insecure disorganized attachment, and low-range synchrony with avoidant attachment (Jaffe et al., 2001). This observation, that synchronization in the mid-range is associated with a more adaptive outcome than at the extremes of very high or very low synchrony, also applies to adult psychotherapeutic interaction (Holtz, 2004). Reich et al. (2014) examined whether synchrony in vocal pitch between psychotherapists and clients is associated with rapport. Their results indicate that pitch synchrony did occur in the sessions, but higher levels of synchrony were related to poorer therapeutic relationships and greater distress.
(d) Interactive synchronization between mother and infant at four months is bidirectional but asymmetrical. Maternal coordination with the infant is higher than infant coordination with the mother (Beebe and Lachmann, 2020). Holtz (2004) reported that in adult psychotherapeutic treatment there is also a bidirectional interactive synchronization, which is asymmetrical. However, in contrast to the asymmetry in mother-infant dyads, in adult psychotherapy the patient’s degree of coordination with the therapist is greater than the therapist’s degree of coordination with the patient (Holtz, 2004). Comparing the synchrony in mother-infant interaction and in adult psychotherapy reveals important parallels, but there are also substantial differences.

Prosocial behavior as an effect of interactive synchrony develops at about 12 months. Tunçgenç et al. (2015) carried out two studies investigating the time window in which the prosocial effects of synchronous movement emerge. It was found that movement synchrony exclusively guides infants’ social choices at 12 months, whereas 9-month-olds did not show any preferences for synchronous movements in social or non-social contexts suggesting that the prosocial effects of movement synchrony emerge toward the end of the first year of life (Tunçgenç et al., 2015). Dancing, singing and music production, which require the ability to entrain to a rhythmic beat, encourage high levels of interpersonal coordination. In 14-month-old infants this prosocial behavior was specifically directed to individuals with whom the infants had experienced synchronous movement (Cirelli et al., 2014). At about 15 months infants have the capacity to make inferences about others’ affiliations based on perceived movement synchrony, and are able to predict social preferences for synchrony from a third-person perspective (Fawcett and Tunçgenç, 2017). This research supports the rather early onset of prosocial behavior in the context of experienced interactional synchrony and the gradual extension of synchrony cognition from a first-person to a third person perspective.

Within the framework of an enactive theory of infant development and language acquisition, early forms of intentionality are considered to arise from a low-level coordination of “moving together” (Ra̧czaszek-Leonardi et al., 2013). The developmental basis of participatory sense-making according to this concept lies in the shaping of as-yet-uncoordinated “individual behaviors into meaningful events through repetitive interactions” (Ra̧czaszek-Leonardi et al., 2013). The question how low-level automatic processes of coordination and synchrony can become meaningful is also a key issue in psychotherapeutic processes. According to Ra̧czaszek-Leonardi et al. (2013) the meaningfulness of low-level procedural interaction is closely related to the interactants shared orientation toward the social context in which the interaction is taking place.

## Synchrony in Psychotherapy

There are several aspects underlining the importance of synchrony research for psychotherapy. Compared to the substantial empirical evidence supporting the predictive value of the therapeutic alliance for psychotherapy outcome in various meta-analyses (Orlinsky et al., 2004; Norcross and Wampold, 2011; Flückiger et al., 2012), relatively little is known about the specific components of the non-verbal communicative behaviors that facilitate the development of the relationship quality. Ramseyer and Tschacher (2011, 2014), referring to the concept of rapport (Tickle-Degnen and Rosenthal, 1990), were the first to demonstrate that non-verbal synchrony is associated with session-level processes and therapy outcome. Altmann et al. (2020) recently reported on a trial including a homogeneous group of patients with social anxiety disorder that, particularly in CBT synchrony in early phases of treatment, predicted improvement in interpersonal relationships. Pacing and leading between patient and therapist were associated with differential effects. The findings, which corroborate the association between movement synchrony and therapy outcome are in agreement with meta-analyses supporting the prosocial effects of synchrony (Rennung and Göritz, 2016; Mogan et al., 2017). Movement synchrony, measured using Motion Energy Analysis (MEA), was suggested as an adjunct to the standard assessment of ruptures within the psychotherapy dyad (Friedman, 2020).

Other recent studies have investigated the relation between movement synchrony and the synchrony of facial affect displays in patients with depression (Altmann et al., 2021). It was found that movement synchrony and synchronous positive facial expressions were diminished in depression. In a sample of patients with anxiety disorder low non-verbal synchrony in the initial phases of treatment was reported as a predictor of premature termination of therapy (Schönherr et al., 2019a).

However, the current evidence on the synchrony-outcome link is not unequivocal (Lutz et al., 2020) investigated the correlation of movement synchrony and early change in psychotherapy. In a sample of 212 patients with mixed diagnoses who underwent cognitive behavioral psychotherapy, three subgroups were identified with regard to early changes in treatment: one subgroup with slow improvement, one with fast improvement and one with early deterioration. In contrast to expectations, the results indicate that lower levels of interpersonal synchrony were related to early response and higher stability of early improvement (Lutz et al., 2020). In a small intervention study using a body therapy approach in patients with schizophrenia, Galbusera et al. (2016) found an inverse relationship between movement synchrony and treatment outcome. Similarly, in a large naturalistic psychotherapy study, Paulick et al. (2018) observed that patients experiencing non-improvement with consensual termination yielded the highest synchrony measures, whereas non-improved patients who dropped out showed the lowest synchrony scores. Reich et al. (2014) studying vocal synchrony reported that in their study higher vocal synchrony was associated with poorer outcome. These findings suggest that more synchrony is not always more beneficial, especially in the field of emotional synchrony (Mayo and Gordon, 2020).

Various studies investigate synchrony at the level of linguistic and vocal aspects of language and speech using different methodologies. Vocal synchrony is often conceived as an indicator of empathy and/or arousal. Bryan et al. (2018) studied the match between patients’ and clinicians’ mean level of emotional arousal during psychotherapy based on the quantification of the vocal “mean fundamental frequencies.” Fundamental frequency is the lowest frequency of a periodic waveform and in music it is perceived as the pitch of a tone. Results show that vocal patient–clinician synchrony was positively correlated with emotional bond ratings (Bryan et al., 2018). Other studies using the mean fundamental frequency as a measure of vocal synchrony reported an association between synchrony and empathy ratings in clinical dyads (Imel et al., 2014).

A linguistic approach to synchrony research in psychotherapy was suggested by Borelli et al. (2019) using Language-style matching (LSM) as a measure to capture the (unconscious) linguistic aspects of interactional synchrony. LSM assesses the similarity in the frequency of use of functional features of language (e.g., pronouns, prepositions, and conjunctions) and thereby aims to shed light on the procedural level of linguistic utterances (Borelli et al., 2019). In a small pilot study of substance dependent women, LSM correlated inversely with interpersonal problems early in treatment (ibd.).

A semantically and content-based approach focusing on synchrony in language use was suggested by Lord et al. (2015). The authors used the software program LIWC (Pennebaker et al., 2007), which allocates word counts to a set of theoretically derived categories. The occurrences of both therapist and patient use of these categories in adjacent talk turn pairs were averaged over all talk turns. Language style synchrony (LSM) turned out to be positively associated with empathy displayed during the sessions (Lord et al., 2015).

In sum the relatively small number of studies on language synchrony in psychotherapy focus either on vocal aspects of speech (e.g., Imel et al., 2014; Tomicic et al., 2017; Bryan et al., 2018) or use semantic (content-)based analytic methods (Borelli et al., 2019). Preliminary evidence reveals a positive association between synchrony (captured by these measures), the experience of empathy and the therapeutic bond.

In linguistic research (but not in psychotherapy research) the role of synchrony for the creation and reinforcement of meaning has been extensively studied. Schober and Clark (1989) and Schober (1993) have argued that synchrony has an effect on the individual enhancement of referential understanding. In a recent contribution, Deppermann et al. (2021) discuss the role of both synchronous and sequential forms of interpersonal coordination for mutual understanding in talk-in-interaction. The potential of prosodic and movement synchrony for collaborative meaning making has been studied for a variety of sequential designs of interaction (cf. Clark, 2005; Deppermann, 2014; Oloff, 2014). Sequences of synchronized, i.e., quasi-simultaneous speech have been investigated in collaborative story tellings (Pfänder and Couper-Kuhlen, 2019; Schmidt, 2020), in greetings (Pillet-Shore, 2012), in phone call closings (Auer, 1990) and in competitive turn-taking (French and Local, 1983; Bolden et al., 2019). Both bodily and verbal alignment have been reported as constitutive for taking (and negotiating) epistemic or affective stances (Lerner, 1992; Fox, 2001; Stivers, 2008; Heritage, 2012; Myers and Lampropoulou, 2012; Sidnell, 2012; Reynolds, 2015; Imo and Lanwer, 2019). Alignment and synchronization have been found to be a constitutive ingredient in almost all of these sequential designs (Deppermann et al., 2021). In other contexts, such as situations of multi-activity (Mondada, 2014a, b) and multi-tasking (Rosen, 2008; Postrel, 2009), interactional synchrony is hampered and often seriously challenged. If it occurs at all in these contexts, it seems to serve mainly as a repair mechanism in order to maintain or restore a fluent interaction.

One of the rare examples of a multimodal mixed method approach to synchrony research in psychotherapy is a single case study reported by Kykyri et al. (2019) in which the linguistic content of the conversation was correlated with posture, movement and autonomic responses (respiration and skin conductance) in order to identify synchrony correlates of the therapeutic relationship. An interesting finding of this study, involving a brief sequence of a couples therapy, is that synchronization processes were observed not only in those individuals that were actively participating in the interaction but also in those passively listening.

While the prosocial effects of synchrony have been extensively studied (Rennung and Göritz, 2016; Mogan et al., 2017), the effects of interactive synchronization on self-perception and self-concept have not been investigated in detail (Galbusera et al., 2019). However, this perspective is relevant when it comes to understanding the consequences of a dysfunction of synchronization ability. It seems plausible that disturbances in interactional coordination may have extremely negative consequences on self-esteem and other mental functions (Lumsden et al., 2014). A clinical focus on synchrony is still in its infancy and there is still little research on how different mental disorders affect the capacity to produce interactional synchrony and vice versa. An exception is the research on movement synchrony in autistic children, where it was found that children with autism spectrum disorder (ASD) exhibited different and less stable patterns of social synchronization ability than controls, and performed motor movements that were slower and more variable in both spacing and timing (Fitzpatrick et al., 2017; Kaur et al., 2018).

## Measuring (Movement) Synchrony in the Clinical Setting

In their review of measures of synchrony in developmental psychology, Leclere et al. (2014) differentiate between three basic approaches to the measurement of synchrony: (1) global interaction scales, (2) synchrony scales, and (3) micro-coded time-series analysis. These three approaches reflect the methodological development of synchrony research in developmental psychology during the last three decades. All assessment tools for synchrony, which are developed through the direct observation of data by trained raters of behavioral coding methods, evaluate the behavior of each partner on a local scale.

Global interaction scales, according to Leclere et al. (2014), assess infant-mother behavior during interaction and also include dyadic parameters. Out of the nine scales listed in this category of global interaction scales (Leclere et al., 2014), four scales integrate dyadic items giving information about the quality of the dyadic interaction without referring directly to synchrony. Two scales use the term synchrony explicitly (the *Coding Interactive Behavior-Scale (CIB)* (Feldman, 2012), and the *Belsky Parent Child Interaction Coding System* (Isabella and Belsky, 1991). The *Coding System for Mother Child Interaction (CSMCI)* (Healey et al., 2010) was designed to assess individual mother and child characteristics, along with the quality of dyadic mother–child interactions. Scales refer both to the individual behavior of the mother (emotionally supportive presence, respect for the child’s autonomy, negative affect, quality of assistance) and the infant (enthusiasm, negativity and hostility, cooperation compliance), as well as to their interaction (affective mutuality, mutual enjoyment, reciprocal interaction). The use of this assessment tool requires trained raters.

The second category, synchrony scales, focuses exclusively on synchrony and dyadic interaction leaving the individual behavior of the interactants aside. Leclere et al. (2014) describe eight scales in this category including the *Bernieri-Scale* (Bernieri et al., 1988) and the *Synchrony Global Coding System* (Skuban et al., 2006). Bernieri et al. (1988), analyzing video clips of mother-infant dyads, used three dimensions to describe the interaction: perception of simultaneous movement; tempo, rhythm similarity; and coordination and smoothness, the Gestalt-like rating of the harmonious meshing of interpersonal behaviors. Both the Bernieri-scale and the Synchrony Global Coding System are based on coder’s perceptions and judgments of synchrony and use *synchrony as a global concept* (Leclere et al., 2014). Other scales are based on a more fine-grained coding of time units, and apply scales referring to specified units of observable interactional behavior, e.g., Taxonomy of Interactional Synchrony (De Mendonca et al., 2011), or Coding Scheme (Mize and Pettit, 1997; Keown and Woodward, 2002).

The third category includes micro-coded time-series analyses. These approaches are mainly quantitative and are based on statistical procedures. Videos of mother-infant interactions are annotated and the two resulting time-series are cross-correlated in order to determine the degree of coherence between the two. The coding itself is often assisted by software systems such as ELAN (Leclere et al., 2014). Micro-coded time-series analysis of synchrony may not only refer to the assessment of behavior but also to behavioral components like acoustic signals or physiological parameters. Global interaction scales and synchrony scales (and to a lesser degree also some of the micro-coded time-series analyses) all are expert-based coding systems using pre-defined categories for the observation and assessment of the participants’ interactive behavior. Expert-based coding approaches share the methodological concern for an optimum of *inter-rater reliability* and *construct validity*. Segmenting and annotating the observed behavior can be difficult. When does a behavior start, and when does it end? “Often, the annotator makes a trade-off because no label accurately describes what he observes” (Delaherche et al., 2012). In addition, the use of manual coding systems is highly time-consuming and tedious.

This prompted the development of *computational methods of assessment*, which are fully automatic and thus more objective. Fully automatic computational assessment tools can be used to capture various aspects of non-verbal synchrony. They aim to measure the degree of similarity between the dynamics of the participants interactive behavior represented by two time-series. The most often applied statistical procedure to analyze movement synchrony is windowed cross-lagged correlation (WCLC) between the two time-series. In this analysis the two time-series within a given time-span (window) are correlated. Next to the zero-order correlation of the time-series, correlations for different time-shifts (lags) between the two time-series can also be computed. Other measures are recurrence analysis or spectral methods (Delaherche et al., 2012). The choice of the length of the window of interaction is of critical importance. If the time-span is too long the assumption of (local) stationarity may be violated; if it is too short cross-correlations between time-series might be underestimated (Boker et al., 2002). Definitions of the length of the window of interaction in the literature vary between 1 s and 10 min with time-lags varying between 0 s and 5 s (Delaherche et al., 2012).

The most common method for the description of movement synchrony, particularly in the context of psychotherapy, is Motion Energy Analysis (MEA). MEA is based on the assessment of differences in sequences of video-frames in recordings of social interaction (Ramseyer, 2010, 2020). The MEA approach captures movement dynamics and is based on the analysis of differences between consecutive frames of a stored sequence (Ramseyer, 2020). For each of the participants’ movement sequences a time-series is generated that represents the time course of the intensity of the individual’s movements. The resulting time-series may be subject to the automated determination of synchrony based on linear time-series analysis methods. Various algorithms are used for the calculation of synchrony based on the correlations of the time-series (Schönherr et al., 2019b). Three output scores can be recorded: average synchrony, maximum of synchrony and frequency of synchrony. The advantages of MEA are the same as for all fully automatic computational assessment tools. The assessment is (a) less time consuming than collecting human ratings, (b) more objective, reliable and valid and (c) does not require the application of additional devices like sensors, etc (Schönherr et al., 2019b). On a conceptual level it can be considered an advantage of MEA that it is focusing on a *systems perspective of interaction*, where synchrony is considered as a property of the interaction dyad (or system), and less as a trait of the interacting individuals.

Several studies have compared human ratings of non-verbal synchrony and non-verbal synchrony obtained by cross-lagged correlation and provided evidence that movements rated by humans and by the algorithms lead to comparable synchrony results (Schmidt et al., 2012; Schönherr et al., 2019b; Fujivara et al., 2020). In a recent study (Feniger-Schaal et al., 2020), the use of windowed cross-lagged correlations (WCLC) with a peak-picking algorithm for assessing differences in leading patterns in the Mirror Game was demonstrated.

Two other automated methods to assess synchrony are motion tracking and motion capture devices (Delaherche et al., 2012). Motion capture devices use sensors which allow for a three dimensional representation of movements. However, their use in psychotherapy is limited because the equipment substantially changes the naturalistic setting of the treatment situation. Studies on single body parts usually use motion tracking devices (for a review see Delaherche et al., 2012). Other more advanced approaches such as OpenPose or VirtualPose are able to accurately track body movements of the human skeleton from a video by means of neuronal network computation without the use of sensors (Huang and Nguyen, 2019).

In the context of studying naturalistic interactions, fully computational measures of synchrony like WCLC are subject to two main criticisms. (1) The interpretation of the results is delicate because it is difficult to know whether what is measured is really synchrony or just a co-occurrence of events without meaning (Delaherche et al., 2012). For MEA a procedure has been established to safeguard the cross-lagged correlations against insignificance by synthesizing surrogate data (pseudo-interactions). Ramseyer (2020) therefore states that the correlations between time-series are non-random. However, this does not mean that the co-occurrence of the interactive behavior between the participants in an interaction is *meaningful*. Local models of the communication dynamics and tools for analyzing what is happening locally during the interaction were required to demonstrate the specific semantic meaning of motion synchrony. (2) WCLC based on the recording of movements (MEA) is essentially focusing on unimodal synchrony. However, many of the prosocial effects of synchrony on the participants of an interaction, as studied in developmental psychology, depend on multimodality. For adult individual psychotherapy the interface between language and multimodal expressive behavior is particularly important. If the focus is on participatory sense-making in the context of psychotherapy, tools for analyzing the semantic content of the interaction in relation to the non-verbal communication are needed. For such a multi-level analysis a combination of computational annotation and coding systems such as OpenPose (Cao et al., 2017; Huang and Nguyen, 2019), OpenFace (Baltrusaitis et al., 2018), OpenSMILE (Eyben et al., 2010) ELAN version 6.0, (Sloetjes, 2021) or Praat (Mayer, 2017) are available. However, even with these computational annotation and coding systems the study of synchronization remains difficult.

## Criteria for the Description of Interactional Synchrony

Current synchrony research has to deal with a considerable conceptual and phenomenological complexity. Wiltshire et al. (2020) state that there is “an overall and pervasive lack of terminological consistency when it comes to referring to interpersonal coordination in psychotherapy” (Wiltshire et al., 2020). The terminological inconsistencies are due to the vast heterogeneity of synchrony phenomena. Therefore in the following paragraphs we suggest a set of criteria that may (a) demonstrate the heterogeneity of possible phenomena and (b) allow for a more systematic approach in the field of synchrony research. The criteria suggested are extracted from empirical studies as well as from theoretical and methodological research on synchrony. Five criteria pertain to the description of synchrony in more general terms; eight other criteria refer specifically to the description of *movement synchrony* (paragraph.6) The list of criteria might not be comprehensive and may be extended as synchrony research evolves. Also their hierarchical order and their potential overlap needs further exploration. However, given these caveats, to the best of our knowledge this is the first attempt to define a set of criteria for a systematization of interactional synchrony phenomena across a wide range of contexts and modalities.

The five general criteria are: (1) context, (2) modality, (3) resources, (4) entrainment, and (5) time-lag.

### Context

Context is conceived here as the specific interactional goal to which the intentional acts of the participants in the interaction are directed. The context does not only shape the form of the interactional phenomena which occur in the dialog, but also their meaning. Synchrony phenomena vary substantially depending on the situational and social context of the interaction in which they emerge (Leander et al., 2012; Paxton and Dale, 2013; Tschacher et al., 2014). Paxton and Dale (2013) demonstrated how different contexts highlighting affiliation or argument determined the interpersonal convergence of body movements. In-phase synchrony decreased significantly during an argument. Synchrony during a problem-solving task differs from synchrony in an interaction aimed at establishing therapeutic alliance (Feniger-Schaal et al., 2020). The social context of the interaction is not necessarily predetermined but may also be co-created by the interactants themselves (Ra̧czaszek-Leonardi et al., 2013), for example in psychotherapy where the specific goals of the interaction may change during the therapeutic process.

### Modality

Modality refers to *categories of expressive behavior*, for example movement, language, gaze etc., similar to the notion of sensory modalities in perceptual psychology. The term is used primarily to distinguish between unimodal and multimodal synchrony or to refer specifically to “crossmodal” synchronization that is synchrony across modalities. A large body of experimental synchrony research is focusing on *unimodal coordination*, for example the coordination of movement OR voice (Mogan et al., 2017). Unimodal synchronization tasks are easier to study. Methods for performing quantitative analyses of relationships across modalities are still scarce (Rohlfing et al., 2020). In natural interaction, however, synchrony is usually *multimodal*, matching rhythm and form across different modalities of expression (Pickering and Garrod, 2006; Louwerse et al., 2012). Cross-modal coordination has been extensively discussed in developmental psychology, where it seems to support the infant’s integration of perception in different sensory modalities (Stern, 1985). There is currently no clear evidence on the differential effects of unimodal versus multimodal coordination in adult interaction.

### Resources

The term resources refers to more specific aspects of expressive behavior. Posture, body sway or head movements, e.g., can be conceived as resources within the modality of movement. The term resources in interactional linguistics is used to denote basic components of social action. The research on synchrony is often focusing either on modalities, e.g., *gaze* (Hadelich and Crocker, 2006; Richardson et al., 2007; Lachat et al., 2012; Stukenbrock, 2014), or on specific resources, e.g., *body sway and posture* (Shockley et al., 2003; Paxton and Dale, 2013), *gestures* (Streeck et al., 2011) *or facial expression* (Peräkylä and Ruusuvuori, 2009). Synchrony that draws from the resources of language and voice appears as congruence in the choice of words (lexical level) or constructions (syntactic level) or with regard to stylistic properties of speech (Giles et al., 2016). Furthermore the quality of the voice: its softness or strength, prosody, gestures, and affect displays, as well as other features of spoken language can all be synchronized and used as resources for mutual interactive adaptation (Imel et al., 2014; Bryan et al., 2018).

Synchrony research in linguistics has been divided into sub-areas investigating the various resources (within the modality of language) of an interactive alignment such as *prosody* (Erickson and Shultz, 1982; Couper-Kuhlen and Selting, 1996; Schönherr, 1997; Couper-Kuhlen, 2002; Keevallik, 2014), forms of *lexical repetition termed* “*conceptual pacts*” (Brennan and Clark, 1996), *syntactic resonance* (Zima and Brône, 2014), *syntactic coordination* (Dale and Spivey, 2006), *syntactic repetition* (Bazzanella, 1996), syntactic *co-adaptation* (Schmid, 2020), or *syntactic alignment* (Wachsmuth et al., 2013).

As with other forms of interactive behavior, the coordination of linguistic utterances has a temporal structure. Auer (2014) conceives projection and latency as two basic principles of spoken language, which enable speakers and recipients to predict structural (and lexical) slots based on what has been said before (projection), or to link emergent syntactic gestalt to that of previous syntactic gestalts (Auer, 2014). Projection is a particularly essential feature of the dialogical co-construction enabling a second speaker to predict (and align with) the next relevant syntactic slot (or in the case of collaboration the next semantic slot) (Auer, 2014). As in movement synchrony, the temporal coordination is also a key feature for linguistic synchrony.

The interplay of speech and bodily movements has been studied in conversation analysis: for example, *multimodal completions* (Oloff, 2014, 2018; Mondada, 2015), *overlap resolution* (Oloff, 2009, 2013), and *responding to requests* (Schmitt, 2004; Rauniomaa and Keisanen, 2012; Rossi, 2015). However, these latter studies have not used the term synchrony, but rather used notions such as “interpersonal coordination” or “alignment” (Schmitt, 2004; Ford et al., 2012; Deppermann, 2013a, b).

### Entrainment

Cacioppo et al. (2014) differentiate between three forms of synchrony according to the different forms of entrainment: reciprocal entrainment (resulting from the intentional synchronization of *all* interactants), unilateral entrainment (where only a single actor is initiating synchronization) and orchestral entrainment (where an external pacemaker is entraining synchrony). Pacing and leading in movement synchrony can be reliably assessed with algorithms using WCLC of movement data (Feniger-Schaal et al., 2020; Ramseyer, 2020). The type of entrainment is intrinsically linked to different forms of synchrony depending on the context. Studies on psychotherapy showed that the outcomes of synchrony led by the therapist and synchrony led by the client are not the same (Ramseyer and Tschacher, 2011; Schönherr et al., 2019a, b).

Related to the issue of entrainment is the question to what extent synchrony should be considered a contingent phenomenon of the two interactants’ spontaneous behavior (without a semantic or propositional content) (Lakin et al., 2003; Pickering and Garrod, 2006; Hove and Risen, 2009), or whether it results from interactional achievement and is due to intentional social cooperation (Richardson et al., 2007, 2009; Ramseyer, 2010; Fusaroli et al., 2014). This differentiation overlaps with the concept of a planned interpersonal coordination (striving toward a common goal) versus an incidentally emerging cooperation (Knoblich et al., 2011). Some forms of mimicry are largely non-conscious and automatic whereas other forms such as co-speech gestures are closely linked to the speech that they accompany both in content and timing and are fully conscious. Bergmann and Kopp (2012) assume that in the alignment of gestures high-level mechanisms in terms of the signaling of links between movement form and meaning, and low-level mechanisms of priming and motor resonance, often co-occur.

### Time Lag

The time-lag defines the time in which the behavior of the interactants is considered to be related. According to Altmann (2013) synchrony phenomena with regard to time-lag can be differentiated into three groups: (1) no time-lag, perfectly synchronous, simultaneous behavior or matching; (2) synchronous behavior with a time-delay, echoing, alignment, imitation, or mimicry (with a time-lag); and (3) convergence, increasing similarity, and adaptation (increasing similarity over time). The definition of the maximum appropriate lag varies between researchers (Schönherr et al., 2019b). Investigation of the coordination of skin conductance level showed a meaningful non-verbal synchrony with a maximum time-lag of 7 s (Robinson et al., 1982). Altmann (2011) used a maximum time-lag of 2.5 s, while Ramseyer and Tschacher (2011) used a time-lag of 5 s. In principle time-lag is an important dimension for the characterization of synchrony phenomena and further research is needed to empirically validate the definition of the appropriate lag. This might vary between signals or modalities. The synchronization of physiological processes might have a time dynamic that differs from the dynamics of synchronization in social, cognitive and perceptual processes (Tschacher et al., 2020).

## Criteria for the Description of Movement Synchrony

Table 1 offers an insight into the complexity of movement synchrony in relation to the different spatial and temporal dimensions of movement. Two systems moving in perfect synchrony should match according to different spatial-temporal dimensions, but to describe complex patterns of synchrony, different dimensions must be taken into account: space, time, flow, and weight (Cipolletta, 2013).

**TABLE 1:**
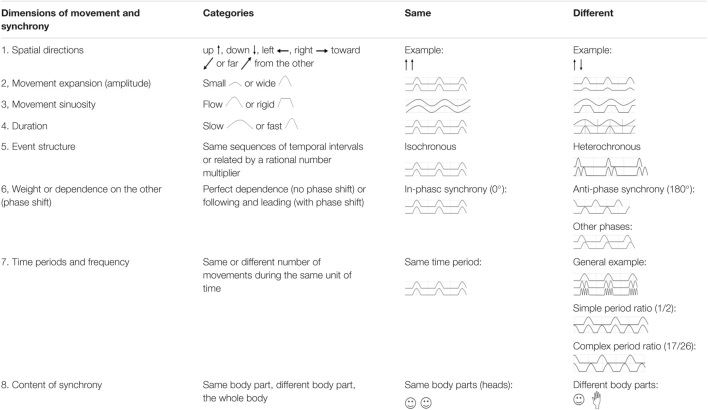
Dimensions of movement synchrony.

Two or more systems could move following the same spatial direction, for example they could both move *up or* both *down*, both toward the *right side or* both toward the *left side*, both *toward or away from the other* (spatial direction, see point 1 of Table 1). The two movements could be qualitatively similar according to their expansion, i.e., *wide or small* movements (amplitude, see point 2 of Table 1), or their sinuosity, i.e., *fluid or rigid* movements (see point 3 of Table 1), or their length/duration, i.e., *slow or fast* movements (see point 4 of Table 1), or the qualitative structure of the movements, both movements could have the same sequence in the same temporal intervals or different sequences related by a rational number multiplier, i.e., *isochronous or heterochronous* (event structure, see point 5 of Table 1) (Ravignani et al., 2014).

Of particular importance for all sorts of dynamic synchrony, including movement synchrony, is the dimension of *phase* (Ravignani et al., 2014) (see point 6 of Table 1). Relative phase is an angle that measures where one rhythm is in its cycle (i.e., its phase) with respect to where another rhythm is in *its* cycle. If two rhythms are in identical parts of their cycles, they have a phase angle of 0° and are in-phase. If two rhythms are in opposite parts of their cycles, they have a phase angle of 180° and are in anti-phase (Schmidt et al., 2012). In-phase indicates that two people are moving at the same time, whereas anti-phase indicates that two people are moving in an alternating fashion (Schmidt et al., 2012). For studies focusing on the timing and the frequency of social coordination, phase is a key parameter (Hale et al., 2020).

Synchronous movements can occur if the number of movements during the same unit of time (frequency) is similar or different (see point 7 of Table 1) (Ravignani et al., 2014). Synchrony can be found between different body parts (see point 8 of Table 1) (Ramseyer and Tschacher, 2016; Ramseyer, 2019). It could occur in a single body part, such as a finger (Oullier et al., 2008), the head (Schmidt et al., 1999; Rienks et al., 2010), a leg (Schmidt et al., 1990) or a specific region of interest (ROI), or the overall movement of the dyad (Delaherche et al., 2012).

Paradigms which have been used to study movement synchrony included *different activities*, e.g., walking, running, moving arms and legs, stepping, arm swaying, waving, finger tapping, rowing, drumming and clapping (Mogan et al., 2017). A first step in the description of movement synchrony should therefore refer to the *dimension of content*: which body parts (and which activities) are involved. The *spatial direction*, the *amplitude*, *sinuosity* and *duration* (velocity) of movements (points 2–4 of Table 1) are often measured using motion tracking devices. Spatial direction, amplitude, movement sinuosity and duration may be the same or different among interactants. If they are the same the overall phenomenology is similar to mimicry or mirroring. If they are different the movement dynamic can still be synchronized: synchrony can be either *identical in pattern and shape (echoing)* (Pickering and Garrod, 2004) or *complementary* (Fusaroli et al., 2014). Dancing for example is based largely on synchrony in terms of complementary movement, and not necessarily on echoing.

Fujiwara and Daibo (2016) suggest spectrum analysis to analyze synchrony in the frequency domain. From the wavelet pictograms, which capture information on the frequencies of the signals recorded during the interaction, the cross-wavelet coherence of the two interactants is calculated, which gives a measure of the coordination of their movements for motion frequency at a given time-point. The cross-wavelet indicates the coherence of the frequency distribution of the interactants over time. Both in-phase and anti-phase movements in this representation are perceived as modes of synchronization in time. Phase and frequency are intrinsically related to the temporal structure of behavioral synchrony.

## Conclusion: Synchrony Research in Psychotherapy

The relevance of synchrony research for psychotherapy lies largely in its potential to enhance the understanding of the procedural dynamics of the therapeutic relationship, which is one of the most important mediators of change and predictors of outcome in therapy (Norcross and Wampold, 2011; Flückiger et al., 2012). Synchrony has been related to the interactional correlates of empathy (Imel et al., 2014) and the therapeutic bond (Bryan et al., 2018), which are both important for the development of rapport. Synchrony research has also introduced a variety of new research methods capturing the different modalities of expressive behavior in interaction [e.g., Motion Energy Analysis (MEA)] (Ramseyer and Tschacher, 2006, 2011). Psychotherapeutic process research makes use of these methods and will extend them to further multimodal interactional studies. With regards to theory, synchrony research underlines a dynamic systems approach to the therapeutic dialog in which the process of participatory sense-making (De Jaegher and Di Paolo, 2007) is a key component. Participatory sense-making is understood as the coordination of intentional activities between interactants (De Jaegher and Di Paolo, 2007; Fuchs and De Jaegher, 2009). To conceive the psychotherapeutic interaction as a dynamic system implies that the system is itself influencing the interaction in addition to, and beyond the participating individuals’ activities and contributions. Within the theoretical framework of embodied cognition (Cuffari et al., 2015) coordination in interaction is a key issue. Drawing from developmental psychology (Nomikou et al., 2016; Ra̧czaszek-Leonardi et al., 2019), artificial intelligence research and linguistics (e.g., Rohlfing et al., 2020), a theory on the intersubjective constitution of meaning is emerging which enables us to conceptualize processes of change in psychotherapy.

The empirical results of synchrony research in psychotherapy, by contrast, have lead to an ambiguous picture and are not conclusive. Some evidence suggests a link, for example between movement synchrony and therapeutic outcome, while other studies report diverging results. There is an agreement that more studies on the synchrony-outcome link in psychotherapy are necessary considering methodological issues like comparable algorithms for synchrony (Schönherr et al., 2019b), homogeneous patient groups, standardized outcome measures, stage of treatment and differential effects of therapists and patients pacing and leading (Altmann et al., 2021). The effects of synchrony may also depend on the modality under investigation. Vocal pitch synchrony for example, as opposed to movement synchrony, may be associated with conflictual social interactions, which in psychotherapy could be related to ruptures in the therapeutic relationship (Reich et al., 2014).

The assumption of a linear relationship between synchrony and prosocial effects is not well founded according to the current state of research, and it could be better replaced by questioning what amount of non-verbal synchrony and which modality might be best to enhance prosociality in a specific context. The concept of a dynamic model of synchronization—according to which two tendencies exist simultaneously in human social interaction, one to synchronize with others and another to move out of synchrony and act independently—seems a more appropriate hypothesis (Mayo and Gordon, 2020).

Another issue concerns the time dynamics and the time-scales of synchrony phenomena. As Nomikou et al. (2016) suggested based on their infant research, synchronization processes entail various time-scales, both moment-to moment adjustment processes and repeated interactions building up to a shared interaction history. It may be hypothesized that synchronization processes in the various modalities of expressive behavior differ in their time dynamic. The coordination of movement for example might follow a different time-scale compared to the coordination of linguistic utterances. Processes of change in psychotherapy spread across modalities might also follow different time-scales.

Only few studies so far have investigated multimodal synchrony processes and their effects on therapeutic outcome. In studies applying a multimodal approach, physiological measures were often used (Wiltshire et al., 2020). Multimodal studies encompassing movement AND language are particularly scarce with only rare exceptions (e.g., Kykyri et al., 2019).

As De Jaegher and Froese (2009) have pointed out, not only new intentions but also new meanings emerge in an interaction. The use of linguistic utterances is embedded and embodied in movement synchrony and the latter can elaborate, extend and shape linguistic meaning. To understand the diverse effects of movement synchrony in solving communicative tasks, specific instances of synchrony have to be analyzed in relation to their semantic context and content. Such communicative tasks may be, for example, the negotiation of dissent in psychotherapy or the delivery of an interpretation (Stukenbrock et al., 2021). Methodologically the analysis of these data requires the integration of tools for linguistic analysis in combination with the respective measures of movement synchrony. In order to enable an in-depth analysis of the semantic context of movement synchrony and its effects in dyadic therapeutic interaction a set of criteria for the classification of the various synchrony phenomena, as suggested in the present article, should facilitate such research approaches.

## Author Contributions

All authors listed have made a substantial, direct and intellectual contribution to the work, and approved it for publication.

## Conflict of Interest

The authors declare that the research was conducted in the absence of any commercial or financial relationships that could be construed as a potential conflict of interest.

## Publisher’s Note

All claims expressed in this article are solely those of the authors and do not necessarily represent those of their affiliated organizations, or those of the publisher, the editors and the reviewers. Any product that may be evaluated in this article, or claim that may be made by its manufacturer, is not guaranteed or endorsed by the publisher.
